# Biogeography and Ecology of Magnaporthales: A Case Study

**DOI:** 10.3389/fmicb.2021.654380

**Published:** 2021-05-06

**Authors:** Jia-Wei Feng, Wei-Ting Liu, Jia-Jie Chen, Chu-Long Zhang

**Affiliations:** Ministry of Agriculture Key Laboratory of Molecular Biology of Crop Pathogens and Insects, Institute of Biotechnology, Zhejiang University, Hangzhou, China

**Keywords:** biodiversity, endophytic fungi, meta-analysis, Poaceae, specificity

## Abstract

The order Magnaporthales belongs to Sordariomycetes, Ascomycota. Magnaporthales includes five families, namely Ceratosphaeriaceae, Pseudohalonectriaceae, Ophioceraceae, Pyriculariaceae, and Magnaporthaceae. Most Magnaporthales members are found in Poaceae plants and other monocotyledonous herbaceous plants ubiquitously as plant pathogens or endophytic fungi, and some members are found in decaying wood or dead grass as saprophytic fungi. Therefore, studying the biogeography and ecology of Magnaporthales is of great significance. Here, we described the biodiversity of endophytic Magnaporthales fungi from Poaceae at three latitudes in China and conducted a meta-analysis of the geography and ecology of Magnaporthales worldwide. We found that Magnaporthales is a dominant order in the endophytic fungi of Poaceae. More than half of the endophytic Magnaporthales fungi have a taxonomically uncertain placement. Notably, few endophytic fungi are grouped in the clusters with known saprophytic or pathogenic Magnaporthales fungi, indicating that they may have saprophytic and parasitic differentiation in nutritional modes and lifestyles. The meta-analysis revealed that most species of Magnaporthales have characteristic geographical, host, and tissue specificity. The geographical distribution of the three most studied genera, namely *Gaeumannomyces*, *Magnaporthiopsis*, and *Pyricularia*, in Magnaporthales may depend on the distribution of their hosts. Therefore, studies on the endophytic fungal Magnaporthales from monocotyledonous plants, including Poaceae, in middle and low latitudes will deepen our understanding of the biogeography and ecology of Magnaporthales.

## Introduction

Magnaporthales belongs to Sordariomycetes, Ascomycota. Thongkantha et al. (2009) first introduced the new order Magnaporthales to accommodate Magnaporthaceae, based on the results of morphology and phylogenetic analysis of the large subunit of ribosomal DNA (LSU) and the small subunit of ribosomal DNA (SSU). Klaubauf et al. (2014) separated *Pyricularia* and its related genera and *Ophioceras* and its related genera from Magnaporthaceae and introduced two separate families, Pyriculariaceae and Ophioceraceae, respectively, to accommodate them, according to morphological observation and two-loci phylogenetic analysis of LSU and the large subunit of RNA polymerase II (*RPB1*). Based on the phylogenetic analysis of representative species and genera of Magnaporthales, Luo et al. (2015a) identified three families in Magnaporthales, including Magnaporthaceae, Pyriculariaceae, and Ophioceraceae, and clarified their nutrition and infection modes. Based on four-loci phylogenetic analysis of LSU, SSU, translation elongation factor 1 (*TEF1*), and the second largest subunit of RNA polymerase (*RPB2*), Maharachchikumbura et al. (2016) accepted three families in Magnaporthales, Magnaporthaceae, Ophioceraceae, and Pyriculariaceae, and placed *Pseudohalonectria* in the genera incertae sedis of Magnaporthale. According to the molecular clock analysis of LSU, SSU, *TEF1*, and *RPB2*, Hyde et al. (2017) suggested that Distoseptisporaceae, with divergence times of 121 million years ago (MYA), should be placed within Magnaporthales. Hongsanan et al. (2017) introduced a new family Pseudohalonectriaceae to accommodate *Pseudohalonectria*, based on the molecular clock analysis of LSU, SSU, *TEF1*, and *RPB2*. Zhang et al. (2018) renewed a time-frame for the evolution of Magnaporthales by analyzing the phylogenomics of representative species and genera of Magnaporthales. Magnaporthales originated 31 MYA, and its pathogenic branch originated 21 MYA. According to a multi-loci phylogenetic analysis of LSU, *RPB2*, and *TEF1*, Luo et al. (2019) introduced a new family Ceratosphaeriaceae in Magnaporthales to accommodate *Ceratosphaeria*, and raised Distoseptisporaceae to a new order Distoseptisporales.

Species of Ceratosphaeriaceae, Pseudohalonectriaceae, and Ophioceraceae are saprophytic fungi growing on decaying wood or dead grass. Ceratosphaeriaceae species grow on decaying wood or dead twigs in terrestrial habitats (Luo et al., 2019). Pseudohalonectriaceae species are saprophytic fungi in submerged rotten wood or twigs (Hongsanan et al., 2017; Luo et al., 2019). Ophioceraceae species are isolated from submerged rotten wood or dead herbs (Shearer et al., 1999).

Pyriculariaceae mainly infect shoots of Poaceae or other monocotyledonous plants, including rice, maize, wheat, bluegrass, and *Digitaria*. Members of the family produce an appressorium, and all are pathogenic fungi (Zhang et al., 2016). The rice blast fungus, *Pyricularia oryzae*, is a model fungus to study the interaction between plants and fungi (Valent, 1990). *P. oryzae* was found to infect not only the shoots of rice plants but also roots of rice (Sesma and Osbourn, 2004; Marcel et al., 2010).

Magnaporthaceae species are reported in all plant tissues, including leaf, stem, and root. Their nutritional modes and lifestyles are complex, and most of them are necrotrophic or hemibiotrophic parasites with a host preference, infecting both Poaceae and Cyperaceae. *Buergenerula, Gaeumannomyces*, *Magnaporthiopsis*, *Nakataea*, *Pseudophialophora*, and *Slopeiomyces* are pathogenic fungi. Among them, the take-all fungus of cereals, i.e., *Gaeumannomyces graminis* (Hernández-Restrepo et al., 2016) and summer patch fungus of turf grass, i.e., *Magnaporthiopsis poae* (Luo and Zhang, 2013) are the two most important pathogens. *Bussabanomyces*, *Falciphora*, and *Pseudophialophora* are endophytic fungi (Yuan et al., 2010; Klaubauf et al., 2014; Luo et al., 2015b, 2017). *Kohlmeyeriopsis* was derived from the dead stem of *Juncus effusus* with unknown nutritional modes and lifestyles (Klaubauf et al., 2014). Notably, *Aquafiliformis* are saprophytic fungi on decaying wood and exist in aquatic habits (Luo et al., 2019). *Plagiosphaera* are saprophytes on dead stem and exist in terrestrial habitats (Song et al., 2019). Species of *Muraeriata* are isolated from bark or wood and exist in terrestrial habitats (Huhndorf et al., 2008).

Healthy plants contain abundant endophytic fungi and many unknown species of endophytic fungi; therefore, more unknown biological functions of endophytic fungi need to be explored (Arnold and Lutzoni, 2007; Rodriguez et al., 2009). Poaceae is the most economically important family among seed plants. It includes the main food crops of humans, such as *Oryza sativa*, *Triticum aestivum*, *Zea mays*, and *Sorghum bicolor* (Kellogg, 2015). The survival and reproduction of Poaceae are positively affected by endophytic fungi. In the past, much attention was paid to clavicipitaceous endophytic fungi, namely *Neotyphodium*/*Epichloë*, which are widely distributed in gramineous plants, primarily in the temperate zone, and play an important role in the resistance of gramineous plants to biotic and abiotic stresses (Clay and Schardl, 2002). However, studies on endophytic Magnaporthales fungi are limited, therefore, studies on the endophytic Magnaporthales fungi associated with Poaceae are of great significance. We studied endophytic fungi from healthy plants of Poaceae growing at three provinces, namely Yunnan, Zhejiang, and Inner Mongolia of China, in the tropical, subtropical, and mid-temperate zones, respectively. A total of 220 strains from 1,821 isolates of endophytic fungi belonged to Magnaporthales, with the relative frequency of 12.1% (Liu et al., 2021). The aims of the present study were: (1) to determine the phylogenetic relationships of endophytic Magnaporthales fungi from Poaceae in three geographic origins using a five-loci sequence, including the internal transcribed spacer of ribosomal DNA (ITS), LSU, DNA replication licensing factor (*MCM7*), *RPB1*, and *TEF1* as well as to analyze the relationship between endophytic Magnaporthales fungi and their host tissues and sites; and (2) to conduct a meta-analysis of the global geographic distribution of the genera of Magnaporthales and the species of *Gaeumanomyces*, *Magnaporthiopsis*, and *Pyricularia*, as well as to analyze the relationships between various species of Magnaporthales and their hosts and tissues.

## Materials and Methods

### Collections and Isolations

Between 2017 and 2018, healthy plant samples of the Poaceae family were obtained from those growing at three provinces, namely Yunnan, Zhejiang, and Inner Mongolia of China, in tropical, subtropical, and mid-temperate zones, respectively. Fungal endophytes were isolated as described elsewhere (Yuan et al., 2010). Briefly, the roots and shoots were scissored to ∼5-cm long fragments, and then tissue fragments were then surface disinfected sequentially with 75% alcohol for 3 min and with 1% (available chlorine) sodium hypochlorite for 10 min. The surface disinfected tissue fragments were scissored to ∼0.5-cm long pieces and then placed on malt extract agar media supplemented with 50 μg/mL ampicillin and streptomycin sulfate. The hyphal tips growing on the edge of the tissue pieces were transferred to a new PDA plate from which the fungal cultures were obtained.

### Fungal DNA Extraction, Polymerase Chain Reaction (PCR) Amplification, and Sequencing

Genomic DNA was extracted from cultures grown on PDA by using a modified protocol suggested by a previous study (Cubero et al., 1999). Five loci, including ITS, LSU, *MCM7*, *RPB1*, and *TEF1*, were amplified and sequenced with the primers ITS 1 and ITS4 (White et al., 1990); LS1 and LR5 (Rehner and Samuels, 1995); MCM7-709 and MCM7-1348 (Schmitt et al., 2009); EF1-728F and EF1-986R (Carbone and Kohn, 1999); and RPB1-Ac and RPB1-Cr (Matheny et al., 2002; Castlebury et al., 2004). The PCR protocols were followed as suggested by a previous study (Zhang et al., 2011).

### Sequence Alignment, and Phylogenetic Analysis

Sequences were initially aligned with MAFFT (Katoh and Standley, 2013) using *Cryphonectria parasitica* EP155 as the outgroup, followed by curing with BMGE (Dereeper et al., 2010). The cured sequence alignments were then concatenated with the SequenceMatrix (Vaidya et al., 2011). Finally, a multi-loci phylogenetic tree with ITS, LSU, *MCM7*, *RPB1*, and *TEF1* was constructed with the iqtree v. 1.6.12 (Minh et al., 2020) by using the maximum likelihood (ML) method. A network of diverse endophytic Magnaporthales fungal species from gramineous plants in different locations and from different tissues were analyzed by using the Cytoscape version 3.6.1.

### Meta-Analysis

Meta-analyses for all known species of Magnaporthales were performed. First, according to sequences of the representative species or genera of Magnaporthales, records with sequence information with Per. ident value ≥ 94.3%, a threshold of filamentous fungi based on the genus level (Vu et al., 2019), were retrieved from GenBank. Then, sequences containing information of accession number, host, and country were screened by using the R version 4.0.2, and the ITS phylogenetic tree were constructed to determine its classification status to the genus. Network diagrams were used to show the relationship among various species of Magnaporthales, and its host and tissue were analyzed as above. Geographical distribution of genera of Magnaporthales and species of *Gaeumanomyces*, *Magnaporthiopsis*, and *Pyricularia* were analyzed in R version 4.0.2.

## Results

### Biodiversity of Endophytic Magnaporthales Fungi From Poaceae

**FIGURE 1:**
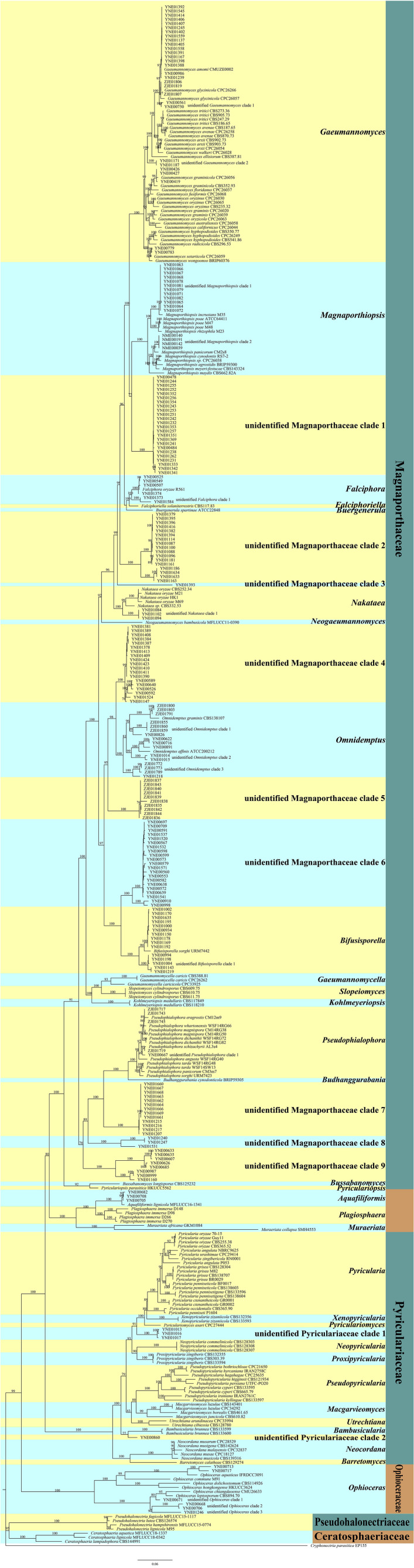
Multi-loci phylogenetic tree based on ITS, LSU, *MCM7*, *RPB1*, and *TEF1* using maximum likelihood (ML).

The retrieved representative sequences used in the study are listed in Supplementary Table 1. The sequences obtained in this study were deposited in the GenBank (accession numbers: MW482542-MW482856, MW478904-MW479096, MW478040-MW478116, MW056504-MW056512, MW055655-MW055657, and MW055631-MW055654). We performed multiple sequence alignments of ITS, LSU, *MCM7*, *RPB1*, and *TEF1* of endophytic fungi of Magnaporthales with those of the representative species of Magnaporthales, and constructed a multi-loci phylogenetic tree with ITS, LSU, *MCM7*, *RPB1*, and *TEF1* (Figure 1) using the maximum likelihood (ML) method. Multi-loci phylogenetic analysis provided a strongly supported clade for families, genera, and species of Magnaporthales.

The multi-loci phylogenetic tree (Figure 1) showed five main clusters corresponding to Pseudohalonectriaceae, Ceratosphaeriaceae, Ophioceraceae, Pyriculariaceae, and Magnaporthaceae. Of the 220 endophytic Magnaporthales strains, six were grouped in a clade containing *Ophioceras* species within the Ophioceraceae cluster, four were grouped in two subclades within the Pyriculariaceae cluster, indicating that they belong to two taxonomically uncertain genera of Pyriculariaceae; and 209 were grouped within the Magnaporthaceae cluster, of which 83 were grouped within eight known genera, namely *Gaeumannomyces*, *Magnaporthiopsis*, *Falciphora*, *Nakataea*, *Omnidemptus*, *Bifusisporella*, *Pseudophialophora*, and *Aquafiliformis*, whereas 126 were grouped in nine subclades, indicating that they belong to nine taxonomically uncertain genera of Magnaporthaceae. More than half of Magnaporthaceous strains we isolated are taxonomically uncertain. Therefore, studies on cryptic Magnaporthaceous endophytic fungi will help understand the biodiversity, biogeography, and ecology of Magnaporthales.

Pseudohalonectriaceae, Ceratosphaeriaceae, and Ophioceraceae are saprophytic fungi. Notably, six endophytic fungal strains in this study belong to *Ophioceras* of Ophioceraceae, thus we speculated that these endophytes could be viaphytes that undergo an interim stage in healthy plant tissues (Nelson et al., 2020). The genera of *Aquafiliformis*, *Muraeriata*, and *Plagiosphaera* reported on decayed wood or dead stems of herbs in aquatic or terrestrial habitats, three endophytic fungal strains in this study belong to *Aquafiliformis* and the genera of *Aquafiliformis*, *Muraeriata*, and *Plagiosphaera* form a separate cluster from Magnaporthaceae. Therefore, we speculated that this branch may be in transition between saprophytic and parasitic. The results may be helpful for us to study the evolution of saprophytic and parasitic fungi in Magnaporthales. Species in Pyriculariaceae are pathogenic fungi. Four endophytic fungal strains in this study, isolated from shoots of healthy plants, belong to two taxonomically uncertain genera of Pyriculariaceae. We speculate that these strains may be pathogenic to plants under the condition of latent infection.

**FIGURE 2:**
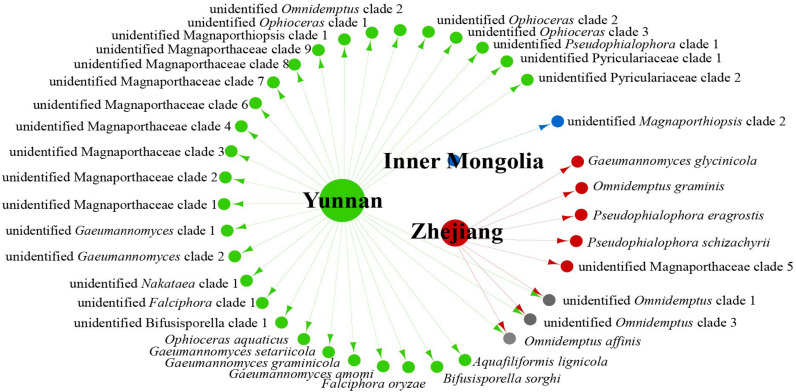
Network of endophytic fungal Magnaporthales species diversity from gramineous plants in Yunnan, Zhejiang, and Inner Mongolia.

**FIGURE 3:**
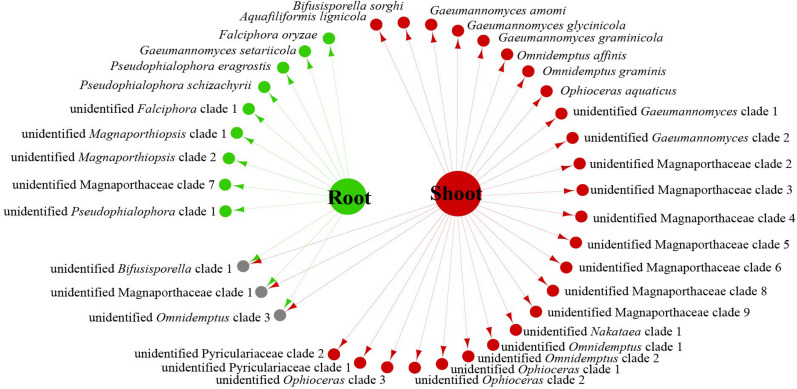
Network of endophytic fungal Magnaporthales species diversity from shoots and roots of gramineous plants in Yunnan, Zhejiang, and Inner Mongolia.

We analyzed the relationship between host tissue and site (Figures 2, 3) and found that *Omnidemptus affinis* and unidentified *Omnidemptus* clade 1 and 3 were collected both in Zhejiang and Yunnan, whereas others were only collected in Zhejiang, Yunnan, or Inner Mongolia. Thus, most Magnaporthales species have characteristics of geographical specificity. Only one species was collected in Inner Mongolia, and most species came from Yunnan and Zhejiang. Moreover, the diversity of species collected in Yunnan was significantly higher than that in Zhejiang and Inner Mongolia (Figure 2). The number of species isolated from shoots of Poaceae is more than that from roots (Figure 3). Unidentified *Omnidemptus* clade 3, unidentified Magnaporthaceae clade 1, and unidentified *Bifusisporella* clade 1 were collected both from the roots and shoots, but the other species were only collected from roots or shoots, indicating that most species have tissue specificity and only a few have extensive colonization in plants.

### Meta-Analysis

By using R, we drew a map of the global geographic distribution of genera of Magnaporthales. A total of 1,378 strains with abundant information were collected by layer screening. Finally, by analyzing the information, we selected 1,378 strains with abundant information of Magnaporthales.

**FIGURE 4:**
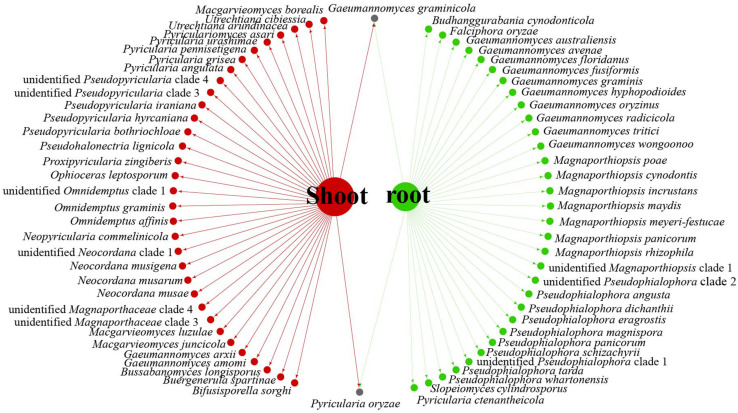
Network of parasitic fungal Magnaporthales species from shoots and roots using meta-analysis.

**FIGURE 5:**
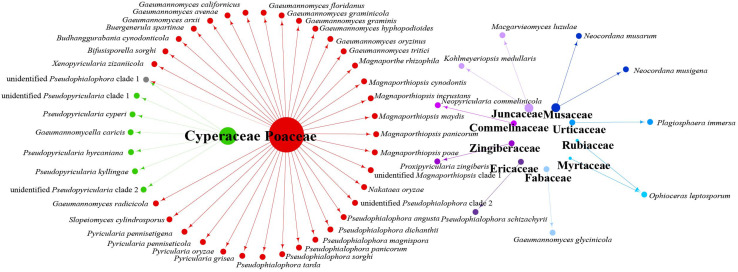
Network of parasitic fungal Magnaporthales species from different host families using meta-analysis.

Data analysis revealed that fungi of Magnaporthales can be collected from the shoot and root, and displayed great diversity in the shoot and root. Most species are collected only from the root or shoot, whereas *Gaeumannomyces graminicola* and *P. oryzae* can be collected from both shoots and roots, which shows that most species have characteristics of tissue specificity (Figure 4). Notably, some endophytic fungal records belong to *Ophioceras* (Figure 4), which was consistent with our investigation (Figure 3). The host range of Magnaporthales is wide, including Poaceae, Cyperaceae, Ericaceae, Juncaceae, Musaceae, Rubiaceae, Commelinaceae, Fabaceae, Myrtaceae, Urticaceae, and Zingiberaceae (Figure 5). The main hosts of Magnaporthales are Poaceae, and the second hosts are Cyperaceae. We speculate that is because Poaceae has been studied more. We found that most species, except unidentified *Pseudophialophora* clade 1 and *Ophioceras leptosporum*, had host specificity. Unidentified *Pseudophialophora* clade 1 uses Poaceae and Cyperaceae as hosts. *Ophioceras leptosporum* uses Myrtaceae and Rubiaceae as hosts.

**FIGURE 6:**
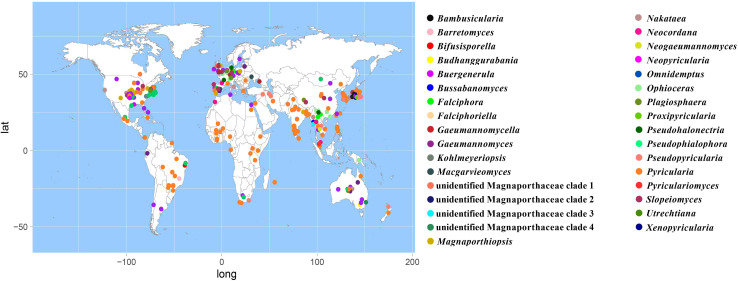
Geographical distribution of Magnaporthales genera using meta-analysis.

**FIGURE 7:**
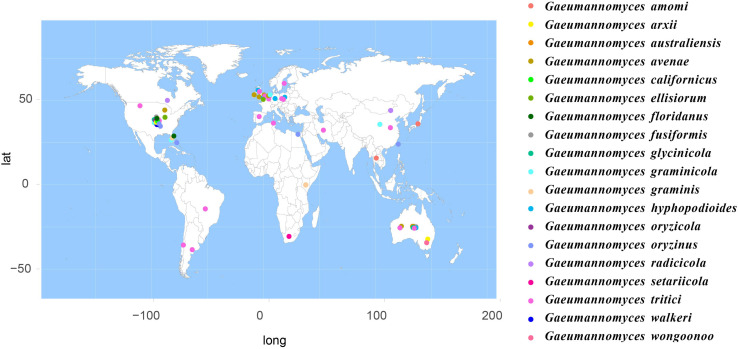
Geographical distribution of *Gaeumanomyces* using meta-analysis.

**FIGURE 8:**
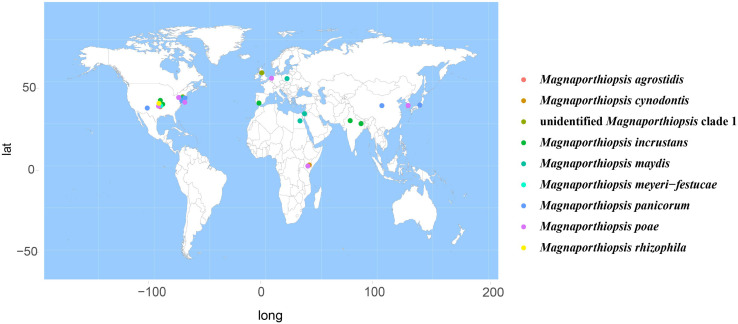
Geographical distribution of *Magnaporthiopsis* using meta-analysis.

**FIGURE 9:**
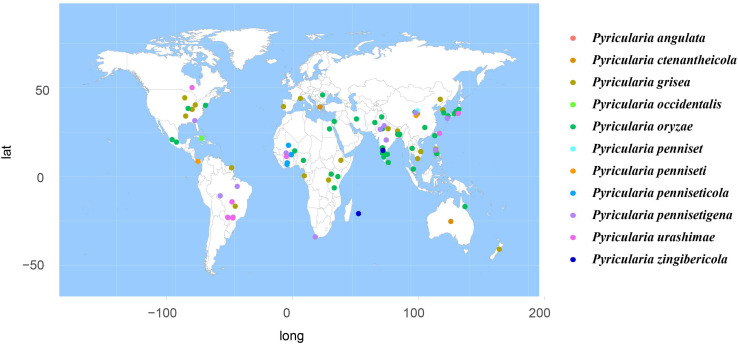
Geographical distribution of *Pyricularia* using meta-analysis.

According to the meta-analysis, strains of Magnaporthales are mainly distributed in middle and low latitudes, with almost none in high latitudes, and the diversity of endophytes in middle latitudes is higher than that in low latitudes. We found that *Gaeumannomyces*, *Magnaporthiopsis*, and *Pyricularia* have a wide distribution range and abundant species (Figure 6), so we mapped the global geographic information map of *Gaeumannomyces*, *Magnaporthiopsis*, and *Pyricularia* to show the distribution of different species in each genus (Figures 7–9). *Gaeumannomyces* and *Magnaporthiopsis* are mainly distributed in temperate regions, whereas *Pyricularia* in tropical and temperate regions. The three genera have characteristics of host specificity (Figure 5), indicating that their geographical distribution depends on their host distribution.

## Discussion

The study of Magnaporthales dates back to the 19th century, when *Nakataea oryzae* (= *Magnaporthe salvinii*) and *Pyricularia grisea* (= *Magnaporthe grisea*) were found to infect rice and *Digitaria* (Thongkantha et al., 2009). Currently, five families, namely Ceratosphaeriaceae, Pseudohalonectriaceae, Ophioceraceae, Pyriculariaceae, and Magnaporthaceae, are accommodated in Magnaporthales with 1, 1, 1, 11, and 22 accepted genera, respectively (Hyde et al., 2020; Wijayawardene et al., 2020). In this study, endophytic Magnaporthales fungi were identified by collecting Poaceae at different latitudes and their taxonomic status was analyzed by phylogenetic analysis. These results indicated that 220 endophytic Magnaporthales strains we isolated were grouped in 20 subclades. Of them, two subclades in Pyriculariaceae and nine subclades in Magnaporthaceae represented presumably novel taxa (genera). As most of the Magnaporthales strains isolated are taxonomically uncertain, further taxonomical analysis is required.

The relationships among the hosts, tissues, and latitudes using a network diagram sum up the information on the biogeographic distribution, host range, and tissues. Meta-analysis indicated that most of the Magnaporthales species possess characteristics of geographical, host, and tissue specificities. However, there were exceptions relative to endophytic fungi in this study. For example, *Gaeumannomyces* was the most studied genus in Magnaporthaceae. *G. amomi* was described as an endophyte from the leaves and pseudostem of herbaceous plants *Amomum siamense* (Zingiberaceae) and *Alpinia malaccensis* (Zingiberaceae) in Thailand (Bussaban et al., 2001); *G. glycinicola* was isolated from the pods of herbaceous plant *Glycine max* (Fabaceae) in Indiana, United States, as a pathogen (Hernández-Restrepo et al., 2016); *G. graminicola* was isolated from the stem base of the grass hosts *Ctenanthe* (Marantaceae) in Netherlands and from *Stenotaphrum secundatum* (Poaceae) and *Eremochloa ophiuroides* (Poaceae) in the United States (Hernández-Restrepo et al., 2016); and *Gaeumannomyces setariicola* was isolated from the host *Setaria italica* (Poaceae) in South Africa (Hernández-Restrepo et al., 2016). In this study, except for two unidentified *Gaeumannomyces* clades (presumably novel species), 17, 3, and another 3 strains belonging to *G. amomi*, *G. glycinicola*, and *G. graminicola*, respectively, were recovered from the shoots of Poaceae plants, and two strains belonging to *G. setariicola* were recovered from the roots of Poaceae plants. These observations indicated that *Gaeumannomyces* species are possibly distributed in herbaceous plant hosts with broader geographical distribution, but exhibit tissue (root and shoot) specificity. For example, in case of *Falciphora* and *Pseudophialophora*, *Falciphora oryzae* (= *Harpophora oryzae*) was first isolated from the roots of wild rice as fungal endophytes (Yuan et al., 2010), four strains belonging to *F. oryzae* and two strains belonging to an unidentified *Falciphora* clades (presumably novel species) were isolated from the roots of three different species of Poaceae (including wild rice) in the Yunnan province in this study. This result indicated that *Falciphora* may be locally distributed in the plant roots of Yunnan province, but in different Poaceae hosts as fungal endophytes. *Pseudophialophora* species were uncovered from the roots of Poaceae grass in the New Jersey Pine Barrens of United States as fungal endophytes (Luo et al., 2014, 2015b). Five strains belonging to *Pseudophialophora eragrostis*, *Pseudophialophora schizachyrii*, and an unidentified *Pseudophialophora* clade 1 (presumably novel species) were recovered from the roots of Poaceae grass in the Zhejiang and Yunnan provinces (Figures 2, 3), and an unidentified *Pseudophialophora* clade 1 was also recovered from the root of Cyperaceae host plant by meta-analysis (Figure 4). It indicated that *Pseudophialophora* species may be specifically colonized in the plant roots and not only in Poaceae plants but also in other monocotyledonous herbaceous plants with global distribution. Therefore, further extensive sampling of the shoots and roots of herbaceous plants, including Poaceae, Cyperaceae, Ericaceae, Juncaceae, Musaceae, Rubiaceae, Commelinaceae, Fabaceae, Myrtaceae, Urticaceae, and Zingiberaceae, in the middle and low latitudes would help better understand the biogeography, ecology, nutritional modes, lifestyles, and evolution of Magnporthales fungi as well as lay a foundation for further study about the interaction between Magnaporthales and plants.

The evolution of fungal pathogenicity was preliminarily revealed by comparative genomic analysis. Saprophytic fungi first evolved into biotrophic pathogenic fungi, and the biotrophic pathogenic fungi evolved into necrotrophic pathogenic fungi and specific biotrophic pathogenic fungi (Spanu, 2012). Magnaporthales exhibit saprophytic and parasitic (pathogenic fungi, endophytic fungi) differentiation in the nutritional modes and lifestyles (Luo et al., 2015a). Differentiation in the nutrition and lifestyles for families of Magnaporthales reflect the evolution of the order Magnaporthales from its ancestors to saprophytic and parasitic fungi (Zhang et al., 2018). Moreover, Magnaporthales exhibit differentiation with root and shoot infections in the infection patterns (Luo et al., 2015a). Pyriculariaceae mainly infects the shoot of Poaceae or other monocotyledonous plants, whereas Magnaporthaceae can infects all portions of Poaceae or other monocotyledonous plants. In our previous study (Xu et al., 2014, 2015), we noted that the endophytic fungi *F. oryzae* (= *H. oryzae*) of Magnaporthaceae originated from pathogenic fungi by comparing the genomic and transcriptomic data. The mechanism of evolution involved changes in the gene composition and expression regulation involved in plant disease resistance responses, autotrophic metabolism, signal transduction, and substance transport. These changes lead to the differentiation of endophytic and pathogenic fungi. Zhang et al. constructed the maximum credibility tree by genome wide analysis, which support that Magnaporthales originated 31 MYA and diverged to different nutritional fungi about 24 MYA, to Poaceae and other monocotyledonous plants about 21 MYA, and to saprophytic fungi on decayed wood in aquatic and terrestrial habitats about 20 MYA and speculated that horizontal gene transfer, secretome and avirulence effector genes, and transposable elements were involved in the differentiation of host or environmental adaptation (Zhang et al., 2018). However, the evolution of saprophytic fungi that live in aquatic or terrestrial habits and parasitic fungi (pathogenic fungi or endophytic fungi) that infect Poaceae or other monocotyledonous plants remain unclear. Thus, the evolution of endophytic and pathogenic fungi also need further analyses. Endophytic fungal species in the clade of *Ophioceras* of Ophioceraceae, the subclade in Magnaporthaceae consisting of *Aquafiliformis*, *Muraeriata*, and *Plagiosphaera* and unknown endophytic fungi in Pyriculariaceae obtained in this study provide the opportunity to study differences in the evolution of saprophytic and parasitic fungi, and pathogenic and endophytic fungi, and to explore the lifestyles and nutritional modes of their common ancestor. In particular, whether there is a gene flow between saprophytic and parasitic fungi, and pathogenic and endophytic fungi, and if this gene migration is unidirectional or bidirectional? These problems are worthy of further exploration.

## Data Availability Statement

The datasets presented in this study can be found in online repositories. The names of the repository/repositories and accession number(s) can be found in the article/Supplementary Material.

## Author Contributions

J-WF performed phylogenetic analysis and meta-analysis, DNA extraction and sequencing, and phylogenetic analysis. W-TL coordinated DNA extraction and sequencing. J-JC coordinated fungal isolation, DNA extraction and sequencing. C-LZ initiated the project, supervised all steps of the experimental work and wrote the manuscript. All authors contributed to the article and approved the submitted version.

## Conflict of Interest

The authors declare that the research was conducted in the absence of any commercial or financial relationships that could be construed as a potential conflict of interest.
