# Supplementary material for: Biogeography and Ecology of Magnaporthales: A Case Study
Source: Front Microbiol. 2021 May 6;12:654380. doi: 10.3389/fmicb.2021.654380 (PMC8134742; doi:10.3389/fmicb.2021.654380)
Supplement: Supplementary file 1 [file Table_1.DOCX]

Table 1 Family, Genus, species, strain number, and GenBank accession numbers of the reference Magnaporthales fungi in this study

| Family | Genus | species | Strain number | ITS | LSU | RPB1 | TEF1 | MCM7 |
| --- | --- | --- | --- | --- | --- | --- | --- | --- |
| Ceratosphaeriaceae | *Ceratosphaeria* | *Ceratosphaeria aquatica* | MFLUCC 18–1337 | MK828612 | MK835812 | / | MN194065 | / |
| Ceratosphaeriaceae | *Ceratosphaeria* | *Ceratosphaeria lignicola* | MFLUCC 18–0342 | MK828613 | MK835813 | / | MN194066 | / |
| Ceratosphaeriaceae | *Ceratosphaeria* | *Ceratosphaeria lampadophora* | CBS 144991 (T) | MN313785 | AY346270 | / | MN313860 | / |
| Magnaporthaceae | *Bifusisporella* | *Bifusisporella sorghi* | URM 7442 (T) | MK060155 | MK060153 | MK060159 | MK060157 | / |
| Magnaporthaceae | *Budhanggurabania* | *Budhanggurabania cynodonticola* | BRIP 59305 (T) | KP162134 | KP162140 | KP162143 | KP162138 | KP162131 |
| Magnaporthaceae | *Buergenerula* | *Buergenerula spartinae* | ATCC 22848 | JX134666 | DQ341492 | JX134720 | JX134692 | JX134706 |
| Magnaporthaceae | *Bussabanomyces* | *Bussabanomyces longisporus* | CBS 125232 (T) | KM484832 | KM484951 | KM485046 | KM009202 | KM009178 |
| Magnaporthaceae | *Falciphora* | *Falciphora oryzae* | R5-6-1 (T) | EU636699 | KJ026705 | KJ026706 | JN857963 | KJ026709 |
| Magnaporthaceae | *Falciphoriella* | *Falciphoriella solaniterrestris* | CBS 117.83 (T) | KM484842 | KM484959 | KM485058 | / | / |
| Magnaporthaceae | *Gaeumannomycella* | *Gaeumannomycella caricicola* | CPC 33925 (T) | MK442584 | MK442526 | / | / | / |
| Magnaporthaceae | *Gaeumannomycella* | *Gaeumannomycella caricis* | CBS 388.81 (T) | KM484843 | KM484960 | KM485059 | KX306674 | / |
| Magnaporthaceae | *Gaeumannomyces* | *Gaeumannomyces amomi* | CMUZE0002 (T) | AY265318 | DQ341493 | / | KX306679 | / |
| Magnaporthaceae | *Gaeumannomyces* | *Gaeumannomyces arxii* | CBS 902.73 | KM484836 | KM484953 | KM485052 | KX306680 | / |
| Magnaporthaceae | *Gaeumannomyces* | *Gaeumannomyces australiensis* | CPC 26058 (T) | KX306480 | KX306550 | KX306619 | KX306683 | / |
| Magnaporthaceae | *Gaeumannomyces* | *Gaeumannomyces avenae* | CBS 187.65 | JX134668 | JX134680 | JX134722 | JX134694 | JX134708 |
| Magnaporthaceae | *Gaeumannomyces* | *Gaeumannomyces californicus* | CPC 26044 (T) | KX306490 | KX306560 | KX306625 | KX306691 | / |
| Magnaporthaceae | *Gaeumannomyces* | *Gaeumannomyces ellisiorum* | CBS 387.81 (T) | [KM484835](http://www.ncbi.nlm.nih.gov/entrez/query.fcgi?cmd=search&db=nucleotide&doptcmdl=genbank&term=KM484835) | [KM484952](http://www.ncbi.nlm.nih.gov/entrez/query.fcgi?cmd=search&db=nucleotide&doptcmdl=genbank&term=KM484952) | [KM485051](http://www.ncbi.nlm.nih.gov/entrez/query.fcgi?cmd=search&db=nucleotide&doptcmdl=genbank&term=KM485051) | KX306692 | / |
| Magnaporthaceae | *Gaeumannomyces* | *Gaeumannomyces floridanus* | CPC 26037 (T) | KX306491 | KX306561 | KX306626 | KX306693 | / |
| Magnaporthaceae | *Gaeumannomyces* | *Gaeumannomyces fusiformis* | CPC 26068 (T) | KX306492 | KX306562 | KX306627 | KX306694 | / |
| Magnaporthaceae | *Gaeumannomyces* | *Gaeumannomyces glycinicola* | CPC 26057 (T) | KX306493 | KX306563 | KX306628 | KX306695 | / |
| Magnaporthaceae | *Gaeumannomyces* | *Gaeumannomyces graminicola* | CBS 352.93 (T) | [KM484834](http://www.ncbi.nlm.nih.gov/entrez/query.fcgi?cmd=search&db=nucleotide&doptcmdl=genbank&term=KM484834) | [DQ341496](http://www.ncbi.nlm.nih.gov/entrez/query.fcgi?cmd=search&db=nucleotide&doptcmdl=genbank&term=DQ341496) | [KM485050](http://www.ncbi.nlm.nih.gov/entrez/query.fcgi?cmd=search&db=nucleotide&doptcmdl=genbank&term=KM485050) | KX306697 | / |
| Magnaporthaceae | *Gaeumannomyces* | *Gaeumannomyces graminis* | CPC 26020 | KX306498 | KX306568 | KX306633 | KX306701 | / |
| Magnaporthaceae | *Gaeumannomyces* | *Gaeumannomyces hyphopodioides* | CBS 350.77 (T) | KX306506 | KX306576 | KM009192 | KM009204 | KM009180 |
| Magnaporthaceae | *Gaeumannomyces* | *Gaeumannomyces oryzicola* | CPC 26063 (T) | KX306516 | KX306586 | KX306646 | KX306717 | / |
| Magnaporthaceae | *Gaeumannomyces* | *Gaeumannomyces oryzinus* | CBS 235.32 | JX134669 | JX134681 | KM485049 | JX134695 | JX134709 |
| Magnaporthaceae | *Gaeumannomyces* | *Gaeumannomyces radicicola* | CBS 296.53 (T) | KM484845 | KM484962 | KM485061 | KM009206 | KM009182 |
| Magnaporthaceae | *Gaeumannomyces* | *Gaeumannomyces setariicola* | CPC 26059 (T) | KX306524 | KX306594 | KX306654 | KX306725 | / |
| Magnaporthaceae | *Gaeumannomyces* | *Gaeumannomyces tritici* | CBS 186.65 | KM484838 | KM484955 | KM485054 | KX306726 | / |
| Magnaporthaceae | *Gaeumannomyces* | *Gaeumannomyces walkeri* | CPC 26028 (T) | KX306543 | KX306613 | KX306670 | KX306746 | / |
| Magnaporthaceae | *Gaeumannomyces* | *Gaeumannomyces wongoonoo* | BRIP 60376 | KP162137 | KP162146 | / | / | / |
| Magnaporthaceae | *Kohlmeyeriopsis* | *Kohlmeyeriopsis medullaris* | CBS 117849 (T) | KM484852 | KM484968 | KM485068 | / | / |
| Magnaporthaceae | *Magnaporthiopsis* | *Magnaporthiopsis agrostidis* | BRIP 59300 (T) | KT364753 | KT364754 | KT364755 | KT364756 | MF178161 |
| Magnaporthaceae | *Magnaporthiopsis* | *Magnaporthiopsis incrustans* | M35 | JF414843 | JF414892 | JF710437 | JF710412 | / |
| Magnaporthaceae | *Magnaporthiopsis* | *Magnaporthiopsis maydis* | CBS 662.82A (T) | [KM484856](http://www.ncbi.nlm.nih.gov/entrez/query.fcgi?cmd=search&db=nucleotide&doptcmdl=genbank&term=KM484856) | [KM484971](http://www.ncbi.nlm.nih.gov/entrez/query.fcgi?cmd=search&db=nucleotide&doptcmdl=genbank&term=KM484971) | [KM485072](http://www.ncbi.nlm.nih.gov/entrez/query.fcgi?cmd=search&db=nucleotide&doptcmdl=genbank&term=KM485072) | / | / |
| Magnaporthaceae | *Magnaporthiopsis* | *Magnaporthiopsis meyeri-festucae* | CBS 143324 (T) | MF178146 | MF178151 | MF178162 | MF178167 | MF178156 |
| Magnaporthaceae | *Magnaporthiopsis* | *Magnaporthiopsis panicorum* | CM2s8 | KF689643 | KF689633 | KF689613 | KF689623 | KF689603 |
| Magnaporthaceae | *Magnaporthiopsis* | *Magnaporthiopsis poae* | M47 | JF414836 | JF414885 | JF710433 | JF710415 | JN993361 |
| Magnaporthaceae | *Magnaporthiopsis* | *Magnaporthiopsis rhizophila* | M23 | JF414834 | JF414883 | JF710432 | JF710408 | JF710384 |
| Magnaporthaceae | *Muraeriata* | *Muraeriata africana* | GKM1084 | / | EU527995 | / | / | / |
| Magnaporthaceae | *Muraeriata* | *Muraeriata collapsa* | SMH4553 | / | EU527996 | / | / | / |
| Magnaporthaceae | *Nakataea* | *Nakataea oryzae* | M21 | JF414838 | JF414887 | JF710441 | JF710406 | JF710382 |
| Magnaporthaceae | *Nakataea* | *Nakataea sp.* | CBS 332.53 | KM484867 | KM484981 | KM485083 | / | / |
| Magnaporthaceae | *Neocordana* | *Neocordana malayensis* | CPC 32837 (T) | MK442593 | MK442533 | / | / | / |
| Magnaporthaceae | *Neocordana* | *Neocordana musae* | CPC 18127 | LN713277 | LN713290 | / | / | / |
| Magnaporthaceae | *Neocordana* | *Neocordana musarum* | CPC 28529 | KY173425 | KY173516 | KY173577 | / | / |
| Magnaporthaceae | *Neocordana* | *Neocordana musicola* | CBS 139316 | LN713285 | LN713286 | / | / | / |
| Magnaporthaceae | *Neocordana* | *Neocordana musigena* | CBS 142624 | KY979749 | KY979804 | KY979886 | / | / |
| Magnaporthaceae | *Neogaeumannomyces* | *Neogaeumannomyces bambusicola* | MFLUCC 11-0390 (T) | KP744449 | KP744492 | / | / | / |
| Magnaporthaceae | *Omnidemptus* | *Omnidemptus affinis* | ATCC 200212 (T) | JX134674 | JX134686 | JX134728 | JX134700 | JX134714 |
| Magnaporthaceae | *Omnidemptus* | *Omnidemptus graminis* | CBS 138107 (T) | MK487758 | MK487734 | / | MK495980 | / |
| Magnaporthaceae | *Pseudophialophora* | *Pseudophialophora angusta* | WSF14RG40 | KP769841 | KP769833 | KP784824 | KP784832 | KP784816 |
| Magnaporthaceae | *Pseudophialophora* | *Pseudophialophora dichanthii* | WSF14RG72 | KP769837 | KP769829 | KP784820 | KP784828 | KP784812 |
| Magnaporthaceae | *Pseudophialophora* | *Pseudophialophora eragrostis* | CM12m9 (T) | KF689648 | KF689638 | KF689618 | KF689628 | KF689608 |
| Magnaporthaceae | *Pseudophialophora* | *Pseudophialophora magnispora* | CM14RG38 (T) | KP769835 | KP769827 | KP784818 | KP784826 | KP784810 |
| Magnaporthaceae | *Pseudophialophora* | *Pseudophialophora panicorum* | CM3m7 (T) | KF689652 | KF689642 | KF689622 | KF689632 | KF689612 |
| Magnaporthaceae | *Pseudophialophora* | *Pseudophialophora schizachyrii* | AL3s4 (T) | KF689650 | KF689640 | KF689620 | KF689630 | KF689610 |
| Magnaporthaceae | *Pseudophialophora* | *Pseudophialophora sorghi* | URM 7423 | KY421938 | KY421941 | KY421944 | KY421942 | / |
| Magnaporthaceae | *Pseudophialophora* | *Pseudophialophora tarda* | WSF14RG48 | KP769840 | KP769832 | KP784823 | KP784831 | KP784815 |
| Magnaporthaceae | *Pseudophialophora* | *Pseudophialophora whartonensis* | WSF14RG66 (T) | KP769834 | KP769826 | KP784817 | KP784825 | KP784809 |
| Magnaporthaceae | *Slopeiomyces* | *Slopeiomyces cylindrosporus* | CBS 610.75 (T) | JX134667 | DQ341494 | JX134721 | JX134693 | JX134707 |
| Magnaporthaceae | *Utrechtiana* | *Utrechtiana arundinacea* | CPC 33994 (ET) | MG934461 | / | MG934473 | / | / |
| Magnaporthaceae | *Utrechtiana* | *Utrechtiana cibiessia* | CBS 128780 (T) | JF951153 | JF951176 | KM485047 | / | / |
| Magnaporthaceae | *Magnaporthiopsis* | *Magnaporthiopsis cynodontis* | RS7-2 | KJ855508 | KM401648 | KP268930 | KP282714 | KP007351 |
| Magnaporthaceae | *Aquafiliformis* | *Aquafiliformis lignicola* | MFLUCC 16-1341 (T) | MK828615 | MK835815 | / | MN194068 | / |
| Magnaporthaceae | *Plagiosphaera* | *Plagiosphaera immersa* | D98 | MN727886 | MN727886 | MN720275 | MN720277 | / |
| Ophioceraceae | *Ophioceras* | *Ophioceras aquaticus* | IFRDCC 3091 | JQ797440 | JQ797433 | / | / | / |
| Ophioceraceae | *Ophioceras* | *Ophioceras chiangdaoense* | CMU 26633 (T) | / | EU571272 | / | / | / |
| Ophioceraceae | *Ophioceras* | *Ophioceras commune* | M91 | JX134675 | JX134687 | JX134729 | JX134701 | JX134715 |
| Ophioceraceae | *Ophioceras* | *Ophioceras dolichostomum* | CBS 114926 | JX134677 | JX134689 | JX134731 | JX134703 | JX134717 |
| Ophioceraceae | *Ophioceras* | *Ophioceras hongkongense* | HKUCC3624 (T) | / | DQ341509 | / | / | / |
| Ophioceraceae | *Ophioceras* | *Ophioceras leptosporum* | CBS 894.70 | JX134678 | JX134690 | JX134732 | JX134704 | JX134718 |
| Ophioceraceae | *Ophioceras* | *Ophioceras aquaticus* | IFRDCC 3091 | JQ797440 | JQ797433 | / | / | / |
| Pseudohalonectriaceae | *Pseudohalonectria* | *Pseudohalonectria fagicola* | MFLUCC 15-1117 | / | KX426219 | / | KX426226 | / |
| Pseudohalonectriaceae | *Pseudohalonectria* | *Pseudohalonectria hampshirensis* | MFLUCC 15-0774 | / | KX426218 | / | KX426224 | / |
| Pseudohalonectriaceae | *Pseudohalonectria* | *Pseudohalonectria lignicola* | M95 | JX134679 | JX134691 | JX134733 | JX134705 | JX134719 |
| Pseudohalonectriaceae | *Pseudohalonectria* | *Pseudohalonectria lutea* | CBS 126574 | MH864160 | JX066706 | / | / | / |
| Pyriculariaceae | *Pyriculariomyces* | *Pyriculariomyces asari* | CPC 27444 | KX228291 | KX228342 | KX228368 | / | / |
| Pyriculariaceae | *Bambusicularia* | *Bambusicularia brunnea* | CBS 133599 | KM484830 | KM484948 | KM485043 | / | / |
| Pyriculariaceae | *Barretomyces* | *Barretomyces calatheae* | CBS 129274 | KM484831 | KM484950 | KM485045 | / | / |
| Pyriculariaceae | *Macgarvieomyces* | *Macgarvieomyces borealis* | CBS 461.65 (T) | MH858669 | DQ341511 | KM485070 | KM009198 | KM009174 |
| Pyriculariaceae | *Macgarvieomyces* | *Macgarvieomyces juncicola* | CBS 610.82 (T) | KM484855 | KM484970 | KM485071 | KM009201 | KM009177 |
| Pyriculariaceae | *Macgarvieomyces* | *Macgarvieomyces luzulae* | CBS 14340 (ET) | MG934440 | / | MG934469 | / | / |
| Pyriculariaceae | *Neopyricularia* | *Neopyricularia commelinicola* | CBS 128303 | KM484868 | KM484982 | KM485084 | KM009199 | KM009175 |
| Pyriculariaceae | *Proxipyricularia* | *Proxipyricularia zingiberis* | CBS 132355 | AB274433 | KM484987 | KM485090 | / | / |
| Pyriculariaceae | *Pseudopyricularia* | *Pseudopyricularia bothriochloae* | CPC 21650 (T) | KF777186 | KF777238 | KY905701 | / | / |
| Pyriculariaceae | *Pseudopyricularia* | *Pseudopyricularia cyperi* | CBS 133595 (T) | KM484872 | KM484990 | AB818013 | / | / |
| Pyriculariaceae | *Pseudopyricularia* | *Pseudopyricularia hagahagae* | CPC 25635 (T) | KT950851 | KT950865 | KT950877 | / | / |
| Pyriculariaceae | *Pseudopyricularia* | *Pseudopyricularia higginsii* | CBS 121934 (T) | KM009164 | KM484991 | KM485095 | / | / |
| Pyriculariaceae | *Pseudopyricularia* | *Pseudopyricularia hyrcaniana* | IRAN 2758C (T) | KP144447 | KP144452 | KY457270 | / | / |
| Pyriculariaceae | *Pseudopyricularia* | *Pseudopyricularia iraniana* | IRAN 2761C (T) | KY457258 | KY457268 | KY457273 | / | / |
| Pyriculariaceae | *Pseudopyricularia* | *Pseudopyricularia kyllingae* | CBS 133597 (T) | KM484876 | KM484992 | KM485096 | / | / |
| Pyriculariaceae | *Pseudopyricularia* | *Pseudopyricularia persiana* | UTFC-PO21 (T) | MH780926 | MH780974 | MH699975 | / | / |
| Pyriculariaceae | *Pyricularia* | *Pyricularia angulata* | NBRC 9625 | AY265322 | / | / | / | / |
| Pyriculariaceae | *Pyricularia* | *Pyricularia ctenantheicola* | GR0001 | KM484878 | KM484994 | KM485098 | / | / |
| Pyriculariaceae | *Pyricularia* | *Pyricularia grisea* | M82 | JX134670 | JX134682 | JX134724 | JX134696 | JX134710 |
| Pyriculariaceae | *Pyricularia* | *Pyricularia occidentalis* | CBS 365.90 (T) | MH862217 | MH873902 | / | / | / |
| Pyriculariaceae | *Pyricularia* | *Pyricularia oryzae* | 70-15 | GCF_000002495.2 | | | | |
| Pyriculariaceae | *Pyricularia* | *Pyricularia oryzae* | CBS 255.38 | KM484889 | KM484999 | KM485109 | / | / |
| Pyriculariaceae | *Pyricularia* | *Pyricularia penniseti* | P1604 | MH619995 | MH807632 | MH633733 | / | / |
| Pyriculariaceae | *Pyricularia* | *Pyricularia penniseticola* | BF0017 | KM484925 | KM485031 | KM485144 | / | / |
| Pyriculariaceae | *Pyricularia* | *Pyricularia pennisetigena* | CBS 133596 | KM484934 | KM485034 | KM485152 | / | / |
| Pyriculariaceae | *Pyricularia* | *Pyricularia urashimae* | CPC 29414 (T) | KY173437 | KY173527 | KY173578 | / | / |
| Pyriculariaceae | *Pyricularia* | *Pyricularia zingibericola* | RN0001 (T) | KM484941 | KM485037 | KM485157 | / | / |
| Pyriculariaceae | *Xenopyricularia* | *Xenopyricularia zizaniicola* | CBS 132356 | KM484946 | KM485042 | KM485160 | KM009203 | / |
